# Response to the Letter on: “An analysis of deficiencies in the data of interventional drug trials registered with Clinical Trials Registry – India”

**DOI:** 10.1186/s13063-019-4011-2

**Published:** 2020-01-07

**Authors:** Mounika Pillamarapu, Abhilash Mohan, Gayatri Saberwal

**Affiliations:** Institute of Bioinformatics and Applied Biotechnology, Bangalore, Karnataka India

Dear Editor,

Thank you for giving us a chance to respond to the Letter by Moulik et al [1].

Our analysis was not an effort at finger-pointing and holding the managers of CTRI to account. It was merely an effort to point to some of the lacunae in the data (and to show an appreciation of the improvement in the data over the years). Similar analyses also have been done on the data hosted by ClinicalTrials.gov, by various groups, including the managers of that database. The authors of the Letter note that, “For any change in a field (even for an obvious error), the CTRI can only send a request (not enforce) to the registrant to update records.” This point is well taken. The CTRI staff have our complete sympathy.

The following individual points were raised in the Letter:

1. “We submit that the application of only automated analysis on registered trial data is likely to lead to errors in interpretation.” We agree. We have made seven specific recommendations, which we stated would prevent various errors. Perhaps a better wording would have been that they would go a long way to prevent these errors.
2. Table 1: The authors of the letter aver that of the 22 trials, only two are foreign (although we had stated that three are). Is it not illogical that trials from which “India” is removed as a country of recruitment still list several sites in India (see, for example, the first two trials in Table 1, that is, CTRI/2007/091/000042 and CTRI/2012/02/002443)? These are the kinds of data that led to the errors that we noted.
3. “...most of the reported “deficiencies” in the article are also likely because of inadequate understanding and misconceptions regarding CTRI data, some of which are highlighted below.”
  We believe the authors are generalizing from the point above (where, in any case, we have stated the problem with the data). We vigorously deny such generalisation. Our methodology is completely transparent, and two reviewers cleared the manuscript. In a 17-page article, with a large number of very detailed supplementary files, we highlighted 16 categories of problems with the data, aside from two other issues. The authors of the Letter have identified no major problems with the work, largely noting where the wording could have been better. We stand by all the analyses not clarified in this response, within the limits of the programs that we have used.
4. “However, a mismatch in the number of sites with ethics approval can be observed because the number of sites in a multicentre trial is a dynamic field, with sites being added and removed over the course of a study.” It is possible that the numbers may be discrepant for this reason. We simply reported on the numbers that we encountered. Remedial measures for displaying more accurate data may be useful.
5. “With regard to missing data, we would like to reiterate that the CTRI software was revised in March 2011, whereupon several new drop-down lists were included. Hence, trials registered prior (1650 registrations) to this revision had missing data for several fields.” In several places in the Discussion, we have made statements such as “In examining error rates over time, it was clear that either registrants have become more careful in providing this data, or registry staff have checked this field more carefully before accepting a trial.” The sharply declining error rates in recent years are testament to the efforts put in by the managers of CTRI, which we have acknowledged.
6. “Regarding principal investigator fields, the authors have noted that 5% of Indian trials and 40% of global trials did not report PI details. As in ClinicalTrials.gov, this field is not a compulsory field in the CTRI because it is over and above the dataset items specified by the WHO and, hence, should not be counted as an error.” This point is well taken. Perhaps it could have been called a “discrepant point”, instead of an “error”, including the reason for it, for those who wish to use the data.
7. “Furthermore, considerable discrepancy, as well as inconsistency, exists in the number of registered trials reported for the CTRI.” We had provided six datapoints on the number of trials in CTRI between 2008 and 2019. Three of these were figures from other publications. The latest three were our own observations. If, as the authors state, these figures are incorrect, then that is an indication of a problem with the search function of CTRI. On the Trial Search page, if one enters “CTRI” as a keyword, the resulting page provides the number of trials in the registry. It seems to have been quite close for the data in 2017, but not for those in 2018 and 2019. This reiterates our report of problems with the search function of the database. Nevertheless, we note that these numbers were reported in the introduction to showcase the recent rapid growth in the number of records in CTRI. Readers would probably be more interested in the trend rather than the precise numbers.
8. “Methodology of “error rates”, provided in additional files, are quite incomprehensible.” Our methodology of calculating error rates has passed peer review, and the corresponding author is happy to explain this if contacted directly.
9. “At the CTRI, we are of the opinion that the title of the article “An analysis of deficiencies in the data of interventional drug trials registered with Clinical Trials Registry - India” is rather misleading and unnecessarily sensational...” It was certainly not our intention to be sensational. Our aim was solely to identify where there could be improvements in the quality of the data or methods of accessing data. We would also like to clarify that in the first version of the manuscript we had not calculated error rates, which we did during revisions at the request of one of the reviewers.
10. “Interestingly, the authors have also published an article on “Some data quality issues at ClinicalTrials.gov” [4], where they have found missing data and variation in names as well “junk” information in the PI field to the tune of 35% of ClinicalTrials.gov records…. If no junk data are present in CTRI (as is our contention), a mention of that fact would have made this a more balanced and impartial article.” In our study of ClinicalTrials.gov, we used the term “junk data” in a very specific way to indicate records in which the names of PIs were replaced by “non-names” such as designations or call centre numbers. In our study of CTRI data, we have presented our limited findings and cannot make any more general comments on whether or not there is junk information in CTRI.

We would like to end with a general thought. When one sets out to conduct such a study, one does not wish to finger-point for the sake of finger-pointing. A large body of literature exists on the efforts made to ensure that trialists register their trials comprehensively and on time; about errors in registry records; and about the reporting of trial results comprehensively, consistently in different fora and on time. That is, to publicly record the data related to trials and their outcomes in a comprehensive and accurate way. This study is part of such a general effort by various stakeholders (and should be taken in that spirit alone). Often such analyses are conducted by the registry managers themselves. Although value exists in independent researchers conducting such studies, the drawback is that they do not have the same intimate knowledge of everything that happens behind the scenes. We would encourage the staff of CTRI (and other registries) to publish accounts of their processes, and list the challenges they have faced or how they have solved them over the years. This would probably be of interest to those who have set up registries more recently or who intend to do so, and would also enlighten independent researchers such as ourselves.

## Data Availability

Not applicable.

## References

[1] Maulik M et al. A response to Pillamarapu M, Mohan A, Saberwal G: An analysis of deficiencies in the data of Interventional drug trials registered with Clinical Trials Registry-India. BMC 2019, 20: 535. 10.1186/s13063-019-3592-0 PMC6712861 31455366

